# Reduced Juvenile Long-Term Depression in Tuberous Sclerosis Complex Is Mitigated in Adults by Compensatory Recruitment of mGluR5 and Erk Signaling

**DOI:** 10.1371/journal.pbio.1001627

**Published:** 2013-08-13

**Authors:** Wyatt B. Potter, Trina Basu, Kenneth J. O'Riordan, Allison Kirchner, Paul Rutecki, Corinna Burger, Avtar Roopra

**Affiliations:** 1 Department of Neuroscience, Medical Science Center, University of Wisconsin–Madison, Madison, Wisconsin, United States of America; 2 Neuroscience Training Program, University of Wisconsin–Madison, Madison, Wisconsin, United States of America; 3 Department of Neurology, William S. Middleton Memorial VA Hospital and University of Wisconsin–Madison, Madison, Wisconsin, United States of America; University of Otago, New Zealand

## Abstract

Tuberous sclerosis complex (TSC) is a multisystem genetic disease that manifests with mental retardation, tumor formation, autism, and epilepsy. Heightened signaling through the mammalian target of rapamycin (mTOR) pathway is involved in TSC pathology, however it remains unclear how other signaling pathways are perturbed and contribute to disease symptoms. Reduced long-term depression (LTD) was recently reported in TSC mutant mice. We find that although reduced LTD is a feature of the juvenile mutant hippocampus, heightened expression of metabotropic glutamate receptor 5 and constitutively activated Erk signaling in the adult hippocampus drives wild-type levels of LTD. Increased mGluR5 and Erk results in a novel mTOR-independent LTD in CA1 hippocampus of adult mice, and contributes to the development of epileptiform bursting activity in the TSC2^+/−^ CA3 region of the hippocampus. Inhibition of mGluR5 or Erk signaling restores appropriate mTOR-dependence to LTD, and significantly reduces epileptiform bursting in TSC2^+/−^ hippocampal slices. We also report that adult TSC2^+/−^ mice exhibit a subtle perseverative behavioral phenotype that is eliminated by mGluR5 antagonism. These findings highlight the potential of modulating the mGluR5-Erk pathway in a developmental stage-specific manner to treat TSC.

## Abstract 2

A mouse model of the human genetic disorder tuberous sclerosis complex fails to undergo developmental down-regulation of mGluR5 expression and activation of Erk signaling, probably contributing to the aberrant plasticity and epilepsy in this disease.

## Author Summary

Tuberous sclerosis complex (TSC) is a genetic disorder that afflicts around 1 in 6,000 people and results from a mutation in one of two genes, TSC1 or TSC2. TSC patients suffer a number of neuronal symptoms including various degrees of autism, mental retardation, and epilepsy, the latter found in more than 80% of cases within the first year of life. In the TSC mutant mice that are used to model the disease, a region of the brain called the hippocampus fails to undergo long-term depression (LTD), a neuronal process that is important for learning and memory. We find that while this is the case in juvenile mutant mice, adult mice appear to have fixed this deficit. The “fix” involves the ramping up of signaling pathways involving mGluR5 and Erk. Although increased mGluR5 and Erk signaling outwardly fixes the problem of diminished LTD in adulthood, it renders the brain insensitive to the cues and inputs that normally work to control LTD. Moreover, the hippocampus in adult TSC mice is prone to seizures and impaired in learning and memory tasks. We find that drugs that target mGluR5 or Erk signaling repair the problems with excitability and learning deficits.

## Introduction

Tuberous sclerosis complex (TSC) is a multisystem autosomal dominant disorder that is characterized by the development of systemic benign hamartomas and cortical tubers, mental disability, autism, and epilepsy [1],[2]. Affecting approximately 1 in 6,000 people, TSC is caused by mutations in either of the tumor suppressor genes *TSC1* or *TSC2*, which result in altered signaling through multiple cellular pathways that impact neurological processes such as nervous system development, neuronal migration, and synaptic function [1],[3],[4]. Epilepsy occurs in approximately 90% of patients and often during the first year of life [1],[2],[5],[6].

TSC1 and TSC2 form a heterodimeric complex (TSC1/2) that receives signals from protein kinase B(Akt) [7]–[9], extracellular signal-regulated kinase (Erk1/2) [10],[11], 5′ adenosine monophosphate activated protein kinase (AMPK) [12], TNFα-IκB kinase β signaling [13], and glycogen synthase kinase-3β (GSK3β) [14]. In this manner, TSC1/2 functions as a signaling node that modulates the activity of the mammalian target of rapamycin signaling complex-1 (mTORC1) [15],[16]. mTORC1 regulates postsynaptic protein translation, and thereby controls activity-dependent plasticity; specifically, long-term potentiation (LTP) [17] and a form of long-term depression (LTD) induced by the group 1 metabotropic glutamate receptor (mGluR1 and mGluR5) agonist, (*S*)-3,5-Dihydroxyphenylglycine (DHPG) [18].

Given its role in synaptic plasticity, work has sought to elucidate a role for mTOR in epileptogenesis and the activity-dependent strengthening of neuronal networks following seizure [19]–[21]. The specific mTOR inhibitor, rapamycin, blocks the development of spontaneous seizures across multiple models of epileptogenesis [20],[22],[23] (but see [24]). Since inhibition of mTORC1 may have therapeutic value in treating TSC [21],[25], pathways that signal to mTORC1 could represent viable avenues for treating TSC.

Recent work by a number of groups shows that juvenile mice (21 d) heterozygous for TSC2 show reduced hippocampal mGluR-LTD magnitude [26],[27]. We find that although juvenile TSC2^+/−^ mutant mice do indeed manifest reduced LTD magnitude, adult mice (2 mo) show no difference in LTD magnitude from wild-type mice. However, this adult mGluR-LTD in TSC2^+/−^ mutant mice is mechanistically distinct from wild-type LTD; adult mGluR-LTD in the TSC2^+/−^ hippocampus is insensitive to mTOR inhibition. Thus, the TSC2 mutant hippocampus utilizes a compensatory pathway to restore LTD magnitude. We report that whereas WT mice show a developmental down-regulation of mGluR5 expression and Erk phosphorylation, TSC2^+/−^ mice maintain a heightened juvenile level of mGluR5 expression through adulthood. Inhibition of mGluR5 or Erk signaling, but not PI3k-Akt signaling, is sufficient to restore mTOR dependence in adult mGluR-LTD in TSC2^+/−^ hippocampus. Additionally, we report an epileptiform bursting phenotype in TSC2^+/−^ CA3 region of the hippocampus induced by prolonged incubation with DHPG. TSC2^+/−^ slices were more likely to develop long synchronous bursts, compared to WT slices—a phenotype that was eliminated by antagonism of mGluR5-Erk signaling.

## Results

### TSC2^+/−^ Hippocampus Exhibits mTORC1-Independent mGluR-LTD

**Figure 1:**
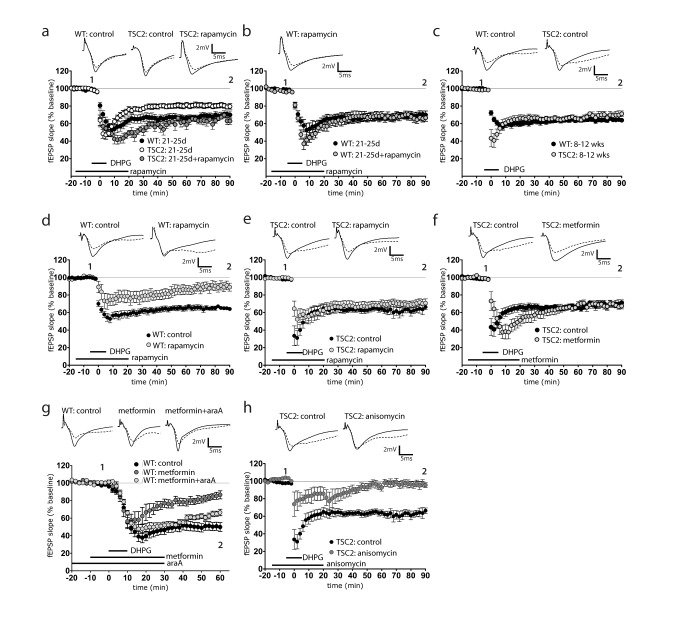
TSC2^+/−^ adult hippocampal slices are unaffected by mTORC1 inhibition. (a) Juvenile (21 d) TSC2^+/−^ hippocampal slices display reduced LTD [79.6±3.1%, *n* = 16(5)] [two-way ANOVA; *F*(1, 26) = 5.72, *p* = 0.024] compared to WT [70.0±4.4%, *n* = 9(4)]. Rapamycin (20 nM) increased LTD magnitude in TSC2^+/−^ slices to WT levels [68.0±3.9%, *n* = 14(5)]. (b) Rapamycin does not impact juvenile WT LTD [*n* = 9(3)]. (c–g) Slices from mature TSC2^+/−^ (8–12 wk). (c) Mature TSC2^+/−^ [*n* = 12(4)] show no difference in LTD magnitude from littermate WT [*n* = 16(5)] controls. (d) Rapamycin (20 nM) reduces mGluR-LTD in mature WT littermates [84.4±5.4%, *n* = 8(4)] [two-way ANOVA; *F*(1, 18) = 17.4, *p* = 0.0006]. (e) Rapamycin (20 nM) does not affect mGluR-LTD in TSC2^+/−^ hippocampus [70.8±5.4%, *n* = 9(5)]. (f) Metformin (5 µM) has no effect on mGluR-LTD in TSC2^+/−^ hippocampus [63.4±8.0%, *n* = 7(3)]. (g) Metformin (5 µM) significantly reduces mTOR-dependent mGluR-LTD in WT slices (metformin, 86.6±4.6%; control, 51.2±5.2%) [two-way ANOVA; *F*(1, 13) = 15.65, *p* = 0.0016], an effect that is reversed with the AMPK inhibitor Ara-A (100 µM) [66.4±6.6%, *n* = 8(4)]. (h) Inhibition of protein synthesis with anisomycin eliminates mGluR-LTD in TSC2^+/−^ hippocampus [96.5±2.5%, *n* = 6(3)] [two-way ANOVA; *F*(1, 13) = 37.62, *p*<0.0001]. Representative traces: solid line is 4 min before DHPG ‘1’, dashed line is at the end of the recording ‘2’.

**Figure 2:**
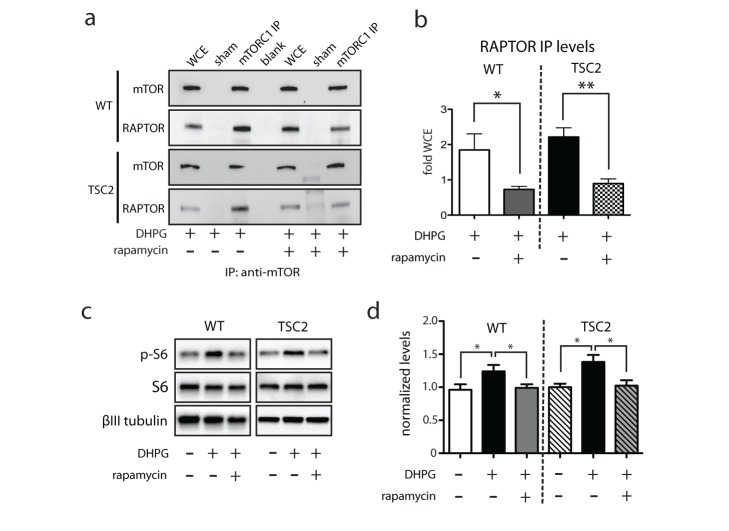
Rapamycin impacts mTORC1 assembly and signaling. (a, b) Rapamycin treatment significantly reduces RAPTOR-mTORC1 association in DHPG-treated WT and TSC2^+/−^ slices (Student *t* test; WT *n* = 4, *p* = 0.030; TSC2^+/−^ *n* = 4, *p* = 0.0043). (c, d) Rapamycin significantly reduces p-S6 levels in WT and TSC2^+/−^ hippocampal slices (Student *t* test; WT *n* = 22, *p* = 0.031; TSC2^+/−^ *n* = 18, *p* = 0.016). Representative traces: solid line is 4 min before DHPG ‘1’, dashed line is at the end of the recording ‘2’. WCE-whole cell extract. *p<0.05, **p<0.005.

In WT hippocampal slices, LTD induced with the group 1 mGluR agonist, DHPG (50 µM, 10 min), requires mTORC1-dependent signaling and protein translation [18],[28]. Similar to recently published reports [26],[27],[29], we found that DHPG induced a lower magnitude of LTD in 21-d-old juvenile TSC2^+/−^ mice compared to WT mice of the same age (Figure 1a). Consistent with the findings of Auerbach et al. [27], the reduced LTD magnitude in TSC2^+/−^ was restored to WT levels with rapamycin. However, we noticed that at 2 mo of age, LTD in mutant mice was nearly indistinguishable in magnitude from WT (Figure 1c). We sought to characterize this unexplored LTD evident in adult 2-mo-old TSC2^+/−^ mutant mice. To assess the role of mTORC1 activity in adult TSC2^+/−^ LTD, we applied rapamycin to TSC2^+/−^ and WT slices. Consistent with the work of Hou and Klann (2004) [18], rapamycin reduced mGluR-LTD in WT slices (Figure 1d). Surprisingly, rapamycin had no effect on mGluR-LTD in adult TSC2^+/−^ slices (Figure 1e). We recently showed that pharmacological activators of AMPK could mimic rapamycin by inhibiting hippocampal mTOR [30]. Thus, we asked whether the AMPK activator metformin could inhibit mGluR-LTD in TSC2^+/−^ slices. Figure 1f shows that, as with rapamycin, metformin also failed to inhibit mGluR-LTD in adult TSC2^+/−^ slices, but was able to inhibit LTD in WT slices (Figure 1g). This inhibition was AMPK-dependent because it could be blocked by the AMPK antagonist, Ara-A (Figure 1g). Since mTORC1 governs postsynaptic protein translation, we tested the possibility that mGluR-LTD in TSC2^+/−^ hippocampus may also be protein synthesis-independent. Treatment with the general translational inhibitor, anisomycin, eliminated LTD (Figure 1h), demonstrating a continued requirement for protein synthesis for LTD expression in adult TSC2^+/−^. Rapamycin works, in part, by reducing the association of mTOR with its obligate partner protein, Regulatory Associated Partner of TOR (RAPTOR), to reduce activation of downstream targets, such as rpS6 [31]. Since rapamycin failed to impact mGluR-LTD in TSC2^+/−^ slices, we tested whether rapamycin was still capable of reducing RAPTOR-mTOR association in TSC2^+/−^ hippocampus. We immunoprecipitated mTORC1 from slices incubated with DHPG for 10 min in the presence and absence of rapamycin. In the presence of DHPG, rapamycin treatment significantly reduced RAPTOR-mTORC1 binding equally well in WT and TSC2^+/−^ slices (Figure 2a and 2b). Additionally, rapamycin significantly reduced p-S6 levels in WT and TSC2^+/−^ slices in the presence of DHPG (Figure 2c and 2d). These data show that in TSC2^+/−^ slices rapamycin is able to disrupt the mTORC1 complex and inhibit downstream signaling to rpS6. Together, these data demonstrate that the adult TSC2^+/−^ hippocampus maintains a requirement for protein translation for the expression of mGluR-LTD yet appears to have altered signaling that circumvents mTORC1 activity.

### TSC2^+/−^ Mice Have Constitutively Elevated mGluR5 and Erk Activation

**Figure 3:**
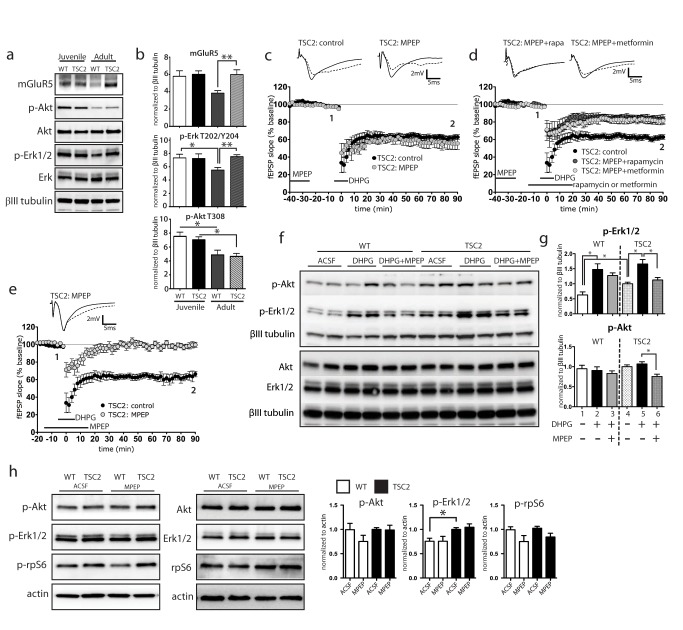
mGluR5 and Erk1/2 signaling is heightened in adult TSC2^+/−^ mice. (a) Acutely harvested hippocampal tissue displays increased mGluR5 expression in TSC2^+/−^ adult mice compared to adult WT mice (one-way ANOVA, *F*(1,70) = 5.536, *p* = 0.0018). There is enhanced Erk1/2 (T202/Y204) phosphorylation in adult TSC2^+/−^ hippocampus compared to WT (one-way ANOVA, *F*(1,74) = 5.859, *p* = 0.0012). There is no observable difference in phosphorylated Akt (T308) between genotypes, however there was a highly significant reduction with age (one-way ANOVA, *F*(1,41) = 6.873, *p* = 0.0007). Western quantifications are displayed in (b). (c) MPEP preincubation (40 µM, present −40 to −20 min), followed by washout does not impact mGluR-LTD magnitude in TSC2^+/−^ hippocampus [MPEP, 55.6±5.7%, *n* = 6(3)]. (d) MPEP preincubation restores rapamycin (20 nM, −10 to 20 min) sensitivity [MPEP+rapamycin, 83.5±5.4%, *n* = 8(3); two-way ANOVA; *F*(1,15) = 21.06, *p* = 0.0004] and metformin sensitivity of mGluR-LTD [metformin+rapamycin, 81.4±4.5%, *n* = 7(3); two-way ANOVA; *F*(1, 14) = 17.15, *p* = 0.0010]. (e) MPEP (40 µM) incubation during LTD induction (present −20 to 20 min) eliminates mGluR-LTD in TSC2^+/−^ hippocampus [99.7±2.7%, *n* = 6(3)]. (f) In WT and TSC2^+/−^ slices, DHPG (50 µM, 10 min) significantly increases phospho-Erk1/2 (one-way ANOVA, *F*(1,55) = 13.1, *p*<0.0001). In WT slices, DHPG increased phospho-Erk1/2 levels (one-way ANOVA, *F*(1,30) = 10.28, *p* = 0.0004). MPEP preincubation, followed by DHPG treatment, causes a significant reduction in p-Akt (one-way ANOVA, *F*(1,60) = 9.402, *p*<0.005), and p-Erk1/2 (one-way ANOVA, *F*(1,55) = 13.1, *p*<0.0001) levels in TSC2^+/−^ hippocampus. (h) 40 µM MPEP (20 min) alone does not impact downstream signaling to Akt, Erk, or rpS6 in WT or TSC2^+/−^ hippocampus. Representative traces: solid line is 4 min before DHPG ‘1’, dashed line is at the end of the recording ‘2’. *p<0.05, **p<0.005.

**Figure 4:**
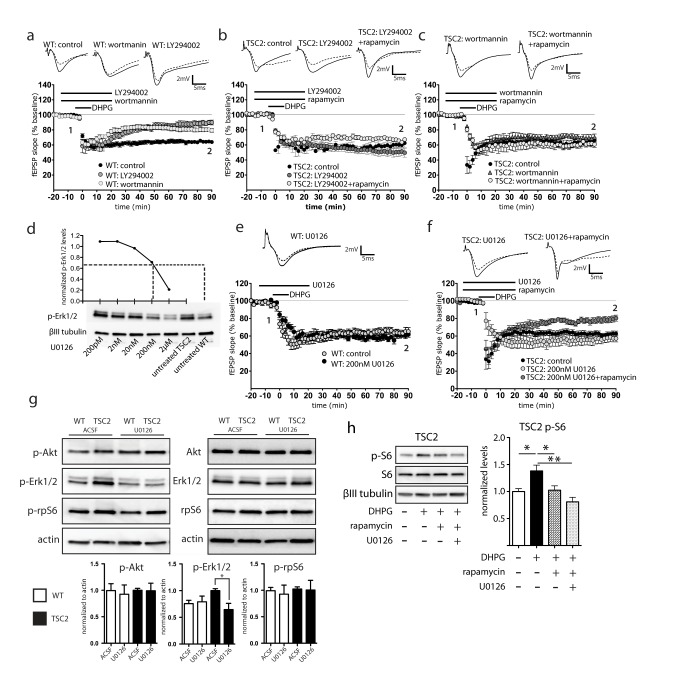
Inhibition of Mek-Erk1/2 signaling, but not PI3K-Akt, reduces mGluR-LTD in TSC2^+/−^ hippocampus. (a) Incubation with the PI3K antagonists LY294002 (10 µM) or wortmannin (500 nM) reduce mGluR-LTD in WT slices [LY294002, 89.9±2.4%, *n* = 9(3); wortmannin, 79.5±3.6%, *n* = 13(3); control, *n* = 16(4)]. (b) LY294002 has no effect in TSC2^+/−^ hippocampus [49.6±5.3%, *n* = 6(3)], nor does it restore rapamycin sensitivity [67.2±1.4%, *n* = 6(3)]. (c) Likewise, wortmannin (500 nM) does not impact mGluR-LTD in TSC2^+/−^ hippocampus [70.4±4.3%, *n* = 7(3)], nor does it restore rapamycin sensitivity [57.4±3.5%, *n* = 7(3)]. (d) Analysis of p-Erk levels in response to a U0126 concentration series indicates that 200 nM U0126 reduces p-Erk1/2 to untreated WT levels. (e) Application of low-dose (200 nM) U0126 does not impact LTD magnitude in WT slices [61.8±5.2%, *n* = 7(3)]. (f) Subtle reduction of Mek-Erk signaling with 200 nM U0126 (−20 to 20 min) does not impact mGluR-LTD magnitude [57.3±4.3%, *n* = 8(3)], yet does restore rapamycin sensitivity [80.4±3.9%, *n* = 7(3), *p* = 0.0054). (g) 200 nM U0126 (20 min) reduces Erk signaling, but does not impact signaling to Akt or rpS6 in WT or TSC2^+/−^ hippocampus. (h) In the presence of DHPG, U0126 blocked activation of rpS6 (Student *t* test; *n* = 18, *p* = 0.016). Rapamycin plus U0126 produced an even greater reduction in p-S6 (Student *t* test; *n* = 15, *p* = 0.0005) (untreated and DHPG-treated quantified data are reproduced from Figure 1). Representative traces: solid line is 4 min before DHPG ‘1’, dashed line is at the end of the recording ‘2’.

In Fragile X Syndrome (FXS), LTD is rendered mTOR-independent by hyperactive mGluR signaling [32]. This observation along with a report of heightened mGluR5 expression in cortical tubers resected from TSC patients [33] prompted us to investigate this receptor in the TSC2^+/−^ mouse. Western blot analysis of acutely harvested hippocampal lysates shows an age-associated decrease in mGluR5 levels in WT lysates. In contrast, mGluR5 expression in older TSC2^+/−^ mice remains at the high juvenile level (Figure 3a and 3b). Thus, adult TSC2^+/−^ hippocampus maintained higher levels of mGluR5 expression over WT, whereas there was no difference in receptor expression in 21-d-old mice. Group 1 mGluR receptors (mGluR1 and mGluR5) activate G_q_-coupled second messenger cascades, which increase PI3K-Akt and Mek-Erk signaling [18],[34]. We therefore compared the activation status of Akt and Erk in acutely harvested WT and TSC2^+/−^ hippocampi (Figure 3a and 3b). As with mGluR5, phosphorylated Erk1/2 (T202/Y204) levels were similar in young WT and mutant hippocampi, but whereas phospho-Erk levels were reduced in adult WT mice, they remained at the high juvenile level in TSC2^+/−^ tissue. Phosphorylated Akt (T308) levels showed an age-dependent decrease in both mutant and WT hippocampi such that there were no differences between genotypes (Figure 3a and 3b). To determine whether heightened mGluR5 signaling contributes to the observed rapamycin-insensitive LTD in adult TSC2^+/−^ hippocampus, we preincubated TSC2^+/−^ hippocampal slices with the noncompetitive mGluR5 antagonist, 2-Methyl-6-(phenylethynyl)pyridine hydrochloride (MPEP). Despite heightened mGluR5 levels in adult TSC2^+/−^, preincubation with MPEP (40 µM for 20 min, followed by a 20-min washout period) had no effect on mGluR-LTD magnitude (Figure 3c). However, when rapamycin was added during the MPEP washout period, we observed a striking restoration of rapamycin-sensitivity such that rapamycin reduced LTD magnitude (Figure 3d). Metformin showed the same inhibition as rapamycin (Figure 3d). Importantly, the level of reduction of mGluR-LTD with MPEP and rapamycin closely resembled the effect of rapamycin alone in WT mGluR-LTD (compare Figures 3d and 1d). Consistent with the work of Hou and Klann (2004) [18] in WT slices, MPEP treatment without washout completely eliminated mGluR-LTD in TSC2^+/−^ slices, demonstrating that mGluR5 signaling is required for the induction of LTD by DHPG (Figure 3e). We observed that pre-incubation with MPEP prevented p-Erk activation in the presence of DHPG in mutants, whereas the same treatment of WT slices did not impact Erk activation (Figure 3f and 3g). MPEP treatment also caused a reduction in Akt activation in TSC2 mutants (Figure 3f and 3g). In the absence of stimulation with DHPG, mGluR5 antagonism with MPEP had no effect on Erk, Akt, or S6 phosphorylation levels in WT or TSC2^+/−^ (Figure 4h).

### Modulation of Mek-Erk Signaling Restores mTORC1 Sensitivity in TSC2^+/−^ Hippocampus

To determine how PI3K-Akt and Mek-Erk signaling might contribute to the mTOR-independent LTD observed in TSC2^+/−^ slices, we applied pharmacological antagonists of PI3K signaling (wortmannin and LY294002), and Mek-Erk signaling (U0126) during LTD induction with DHPG. In accordance with previous studies [18], the expression of LTD was significantly reduced with LY294002 and wortmannin in WT slices (Figure 4a). In contrast to WT, antagonism of PI3K-Akt signaling in adult TSC2^+/−^ slices had no effect on the expression of LTD, nor did it restore rapamycin sensitivity (Figure 4b and 4c). This demonstrates that PI3K inhibition does not reproduce the effects of mGluR5 inhibition in TSC2^+/−^. We hypothesized that the hyperactive Erk signaling was bypassing or short-circuiting the mTOR pathway to drive LTD and render it mTOR-independent and rapamycin insensitive. To address this, we first performed a dose response of U0126 to find a concentration that would “dial down” Erk signaling to untreated WT levels and then assessed rapamycin sensitivity in TSC2^+/−^ slices. From the concentration series of U0126 in TSC2^+/−^ slices, we determined that a relatively low-dose (200 nM) U0126 reduced p-Erk in TSC2^+/−^ slices to untreated WT levels (Figure 4d). If reducing Erk activity is sufficient to restore rapamycin sensitivity in TSC2^+/−^ slices, then reduction of p-Erk to WT levels would be expected to leave mGluR-LTD unaffected, yet render slices responsive to rapamycin. Application of 200 nM U0126 alone did not affect mGluR-LTD magnitude in WT (Figure 4e) or TSC2^+/−^ slices (Figure 4f). However, addition of rapamycin was now capable of reducing mGluR-LTD in TSC2^+/−^ slices (Figure 4f). These data indicate that the subtle reduction of Mek-Erk signaling was sufficient to restore a WT-like response to rapamycin and recapitulate the effects of preincubated MPEP on mGluR-LTD in TSC2^+/−^ slices (compare Figure 3d and Figure 4f). Western blot analysis shows that low-dose 200 nM U0126 alone did not directly impact mTOR signaling downstream to rpS6 (Figure 4g). In the presence of DHPG, however, U0126 prevented activation of rpS6, an effect that was enhanced with rapamycin (Figure 4h). These observations, along with the elevated levels of mGluR5 and phospho-Erk in TSC2^+/−^ slices, support the hypothesis that the aberrant plasticity in TSC2^+/−^ slices arises from heightened mGluR5 and Erk signaling.

### mGluR5 Antagonism Reduces Epileptiform Activity in TSC2^+/−^ Hippocampus

Epilepsy occurs in approximately 80%–90% of individuals with TSC often presenting in the first year of life [3],[35]. Epileptiform bursting activity can be induced in hippocampal slices with prolonged activation of group 1 mGluRs with DHPG. This alteration in excitability persists for hours after the removal of DHPG, is protein synthesis-dependent, and represents an enduring change in network excitability that mimics seizures [36]–[38]. Due to our observation that mGluR5 expression is heightened in the TSC2^+/−^ hippocampus, we reasoned that TSC2^+/−^ slices should be more susceptible to the development of DHPG-induced epileptiform bursting. To test this, we measured field activity in CA3 stratum pyramidale in TSC2^+/−^ and WT slices following DHPG treatment (50 µM, 30 min) and quantified burst number and duration in slices that showed spontaneously occurring synchronous activity. Ictal epileptiform activity was defined as synchronous activity of greater than 2 s with intraburst frequencies of 2 Hz or greater. Interictal epileptiform activity was defined as spontaneously occurring synchronous activity with a duration of less than 2 s.

**Figure 5:**
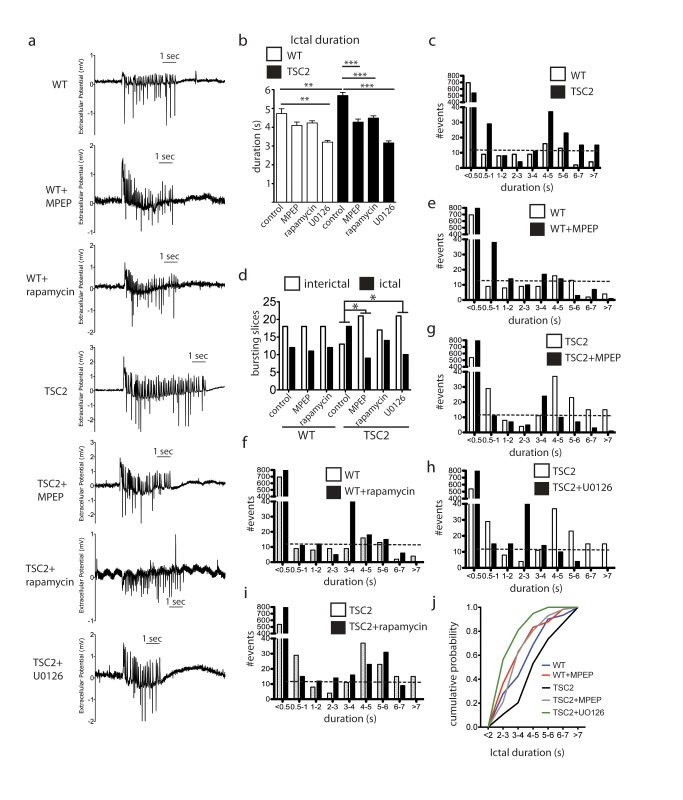
Antagonism of mGluR5 or Erk signaling reduces induction of epileptiform activity in TSC2^+/−^ CA3 hippocampus. (a) Representative traces of WT and TSC2^+/−^ slices following DHPG treatment (50 µM, 30 min) and in the presence and absence of drug. (b) Ictal burst duration is greater in TSC2^+/−^ hippocampus compared to WT (Student *t*-test, *p* = 0.0020) and is significantly reduced with MPEP (*p*<0.005) and U0126 (*p*<0.0005). (c) TSC2^+/−^ slices develop significantly more long-duration bursts in CA3 neurons than WT slices (Chi-square test for trend, *p*<0.0001). (d) Antagonism of mGluR5 (40 µM MPEP) or Mek-Erk (20 µM U0126) signaling during DHPG treatment significantly reduces epileptiform bursting in TSC2^+/−^ slices (TSC2 versus TSC2+MPEP Chi-square, *p* = 0.027; TSC2 versus TSC2+U0126 Chi-square, *p* = 0.041). WT bursting was not significantly different with MPEP (Chi-square, *p* = 0.871). (e) Co-incubation of MPEP and DHPG in WT slices produced an insignificant trend toward less bursting in WT slices (Chi-square for trend, *p* = 0.182). (f) Rapamycin (20 nM) did not impact DHPG-induced bursting in WT slices. (g) MPEP (40 µM) treatment during DHPG incubation significantly reduces CA3 bursting in TSC2^+/−^ slices (Chi-square for trend, *p*<0.0001). (h) U0126 (20 µM) significantly reduces CA3 bursting profile in TSC2^+/−^ slices (Chi-square for trend, *p*<0.0001). (i) Rapamycin (20 nM) did not impact the bursting profile in TSC2^+/−^ slices (Chi-square for trend, *p* = 0.211). (j) Cumulative probability of developing long duration ictal activity is greatest in TSC2^+/−^ slices and decreases with MPEP and even more so with U0126. *p<0.05, **p<0.005, ***p<0.0005.

One hour after removal of DHPG, TSC2^+/−^ slices produced significantly longer burst durations (Figure 5a and 5b) and displayed more long-duration ictal events compared to WT (Figure 5c). In WT slices, MPEP or rapamycin treatment during the DHPG incubation did not significantly impact ictal burst duration (Figure 5b), nor did they affect the development of epileptiform activity (Figure 5d–f). However, in TSC2^+/−^ slices MPEP caused a significant reduction in both the duration of ictal bursts (Figure 5b) as well as the proportion of slices that developed ictal activity (TSC2^+/−^: 58.1% DHPG versus 30.0% DHPG+MPEP) (Figure 5d). Incubation of WT or TSC2^+/−^ slices with 20 µM U0126 produced an even greater reduction in average bursting duration than MPEP (Figure 5b). MPEP and U0126 produced a similar and significant reduction in the proportion of ictal slices (Figure 5d) and each caused a dramatic shift in the bursting profile in TSC2^+/−^ slices toward shorter duration bursts (Figure 5g and 5h, respectively). Overall, rapamycin was less effective at reducing CA3 bursting. Although rapamycin did reduce the average burst duration in TSC2^+/−^ slices (Figure 5b), rapamycin had no statistical effect on the proportion of interictal and ictal slices (Figure 5d), nor did it alter the bursting profile (Figure 5i). The CA3 bursting data demonstrate that mGluR5 and Erk signaling is involved in the development of epileptiform activity in TSC2^+/−^ CA3 hippocampus in response to DHPG, and that the enhanced epileptogenic potential of TSC2^+/−^ slices can be blocked with mGluR5 or Erk antagonism. In summary, the cumulative probability curve shows that addition of MPEP or U0126 to mutant slices reduces the 50th percentile burst duration from 4–5 s to 3 s or 2 s, respectively (Figure 5j).

### TSC2^+/−^ Mice Display a Perseverative Behavioral Phenotype That Can Be Corrected by MPEP

**Figure 6:**
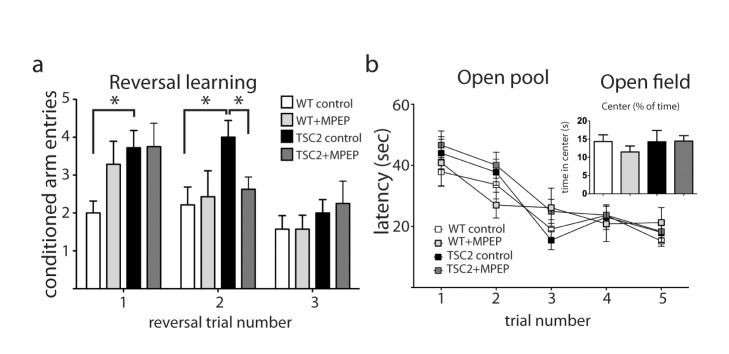
MPEP eliminates a perseverative behavior in TSC2^+/−^ mice. (a) During reversal training, TSC2^+/−^ mice make significantly more errors toward the conditioned arm in the RAWM (two-way ANOVA; WT *n* = 21; TSC2 *n* = 23; *F*(3, 43) = 9.160, *p* = 0.0034). Differences between individual groups were assessed with paired Mann Whitney *t* test: Trial 1, WT versus TSC2; *p* = 0.049. Trial 2, WT versus TSC2, *p* = 0.010; TSC2 versus TSC2+MPEP; *p* = 0.0466. (b) There were no differences for open field performance between genotypes (WT *n* = 14; TSC2 *n* = 12; TSC2+MPEP *n* = 14). There were no differences for open pool performance between genotypes. (WT *n* = 13; TSC2 *n* = 12; TSC+MPEP *n* = 14). NS, not significant, *p<0.05, **p<0.005.

In a previous report, Ehninger et al. (2008) analyzed cognitive and stress-related behaviors associated with tuberous sclerosis in TSC2^+/−^ mice. They reported that TSC2^+/−^ mice display cognitive and stress-related deficits that were corrected by rapamycin [39]. We sought to extend the behavioral analysis to other characteristic behaviors found in tuberous sclerosis, namely autistic perseverative behavior, and to determine if these behaviors in TSC2^+/−^ mice could be due to heightened mGluR5 signaling. We used the radial arm water maze (RAWM) followed by a reversal training protocol to analyze the behavioral phenotype of these mice. After WT and TSC2^+/−^ mice that were administered MPEP or vehicle acquired the hidden platform (Figure S1), the platform was moved to test reversal learning. In the first and second reversal trials, TSC2^+/−^ mice visited the target arm where the hidden platform had been previously placed in trials 1–30 significantly more frequently than WT mice (*p* = 0.0064). This preservative behavior was corrected in TSC2^+/−^ mice injected with MPEP (Figure 6a—Reversal Trial 2; main effect of group: *F*_(1,21)_ = 1.98, *p* = 0.1747). By the third trial, all groups behaved the same. This suggests that mGluR5 contributes to the behavioral phenotype in TSC2^+/−^ mice. Additional behavioral testing revealed no deficits in sensory, exploratory, or motor performance. There were no differences in the performance in the open pool task between any of the experimental groups (Figure 6b; *F*_(3,27)_ = 0.7313, *p*>0.05). Locomotor activity was unaffected by both genotype and treatment as measured in the Open Field task. There were no differences in anxiety and exploration between the experimental groups, as measured by the percentage of time spent in the center (Figure 6b, inset; one way ANOVA *F*_(3,28)_ = 0.47, *p*>0.05).

## Discussion

While the role of dysregulated mTORC1 signaling in the pathophysiology of TSC has been demonstrated previously [27],[39]–[41], our work implicates heightened mGluR5 and Erk signaling as a key component of the irregular plasticity and pathological phenotypes in a model of TSC. Consistent with findings by others, we find that juvenile TSC2^+/−^ mice (21 d old) have reduced mGluR-LTD compared to littermate wild-types [26],[27]. However we show that adult TSC2^+/−^ mice (2 mo) have similar LTD magnitude to age-matched WT mice. This TSC2^+/−^ adult mGluR-LTD is mTOR-independent and thus distinct from WT mGluR-LTD. We show that adult TSC2^+/−^ mice have increased levels of hippocampal mGluR5 expression as well as overactivation of Mek-Erk signaling. Inhibition of mGluR5 signaling with MPEP blocked adult mGluR-LTD in TSC2^+/−^ CA1 hippocampus (Figure 3e). We illustrate, for the first time, that the TSC2^+/−^ hippocampus displays an increased expression of epileptiform activity following prolonged group I mGluR activation, and that this bursting activity can be suppressed via mGluR5 inhibition with MPEP or inhibition of Mek-Erk signaling with U0126 (Figure 5). Finally, MPEP corrected a perseverative behavioral phenotype in TSC2^+/−^ mice, which may be a correlate of an autistic-like behavioral aspect in TSC2^+/−^ mice (Figure 6). These findings suggest that modulation of mGluR5 signaling can correct two major pathological aspects of TSC, namely epilepsy and cognitive dysfunction.

Our work is the first to show a developmental change in mGluR-LTD in a mouse model of TSC. We find LTD magnitude increases in TSC2 mutants from 21 d to 2 mo of age. In contrast, the magnitude of LTD is invariant between juvenile and adult WT mice. Thus it appears that a developmental compensation occurs in mutants to restore a wild type-like LTD. Although mGluR-LTD magnitude is indistinguishable between WT and TSC2 mutant adult mice, we find that the adult LTD in TSC2^+/−^ mice is mechanistically distinct from that of wild-type littermates: mutant adult LTD is rapamycin insensitive and independent of mTOR signaling. Rapamycin insensitivity is not due to a loss of mTOR signaling *per se* or loss of the rapamycin sensing protein FKBP12 because TSC2^+/−^ slices still exhibit rapamycin-dependent loss of RAPTOR/mTOR binding (Figure 2) and inhibition of 1XTheta Burst LTP (Ehninger et al. [39]), suggesting rapamycin sensing is still intact.

Loss of mTOR dependence in TSC2^+/−^ renders LTD nonresponsive to cues, signaling cascades, and inputs that modulate plasticity via mTOR. AMPK is a modulator of mTOR signaling that couples metabolism to plasticity [30]. We have previously shown that AMPK modulates hippocampal LTP and others have demonstrated a role for AMPK in learning and memory through mTOR [42]. Here we show for the first time that AMPK negatively regulates mGluR-LTD in WT mice, suggesting that energy availability controls not just LTP and learning and memory but also LTD in WT mice. Consistent with TSC2^+/−^ adult mGluR LTD being mTOR independent, we find that unlike in wild-type slices, mutant slices display an LTD that is insensitive to metformin and 2DG, well-characterized AMPK activators (Figure 1 and Figure S2). Thus a pathway that regulates plasticity via mTOR in wild-type mice is unable to control this type of plasticity in TSC2^+/−^ mice. Immunity of mutant LTD to mTOR-dependent inputs may underlie some of the pathological phenotypes seen in TSC patients.

The lack of mTOR dependence prompted us to assess whether mGluR-LTD in adult mutant mice still required *de novo* protein synthesis, as is the case for wild-type mice [28]. Our observed mutant LTD is clearly protein synthesis-dependent at 2 mo of age (Figure 1), whereas Auerbach et al. describe a protein synthesis-independent LTD [27] in these mice at 1 mo. Together, these findings suggest that TSC2 mutants transition from a protein synthesis independent to dependent mGluR-LTD as they age.

Interestingly, we find that whereas adult WT mice display a rapamycin-sensitive mGluR-LTD in line with work of many others [18],[28], juvenile WT mice display a rapamycin-insensitive mGluR LTD (unpublished data). Auerbach et al. observed a similar rapamycin-insensitive LTD in juvenile WT slices. Thus, there appears to be a developmental switch from a rapamycin insensitive to sensitive LTD and so reconciles the disparate findings between multiple groups regarding the role of mTOR in hippocampal mGluR LTD. This finding correlates with observations of protein synthesis-independent LTP in juvenile rats [43], which becomes protein-synthesis-dependent in adulthood [44].

Compared to adult wild type, TSC2 mutant hippocampus shows constitutively high p-Erk and increased expression of mGluR5 that are necessary for adult LTD (Figures 3 and 4). Chevere-Torres et al. demonstrated that mGluR-LTD in ΔRG TSC2 mutant mice is also driven by aberrant Erk signaling [26]. Auerbach et al. saw no increase in Erk signaling in young TSC2^+/−^ mice. Similarly, we find that there is no difference in Erk activity between mutant and WT hippocampi at 21 d of age (an age analyzed by Auerbach et al.). Heightened Erk activity in mutant mice is only evident in adult hippocampus and appears to be due to failure of mutants to reduce p-Erk from juvenile levels (Figure 3). These findings would appear to reconcile differences in findings from multiple groups and highlights the importance of developmental stage in neuronal signaling.

We hypothesize that heightened mGluR5 and Erk signaling bypasses mTOR signaling and so renders LTD insensitive to signals that converge on mTOR. Brief preincubation of MPEP, followed by washout, did not by itself inhibit mGluR-LTD magnitude in TSC2^+/−^ hippocampus, but instead restored rapamycin sensitivity to mGluR-LTD (Figure 3d). This suggests that the adult TSC2^+/−^ hippocampus is capable of “wild-type”-like responses if signaling downstream of mGluR5 or attenuation of Erk signaling can be attenuated, and thus opens up a novel therapeutic avenue. The mechanisms driving up-regulation of mGluR5 are not known, and we did not detect a difference in mGluR5 transcript between WT and TSC2^+/−^ hippocampus (unpublished data). This suggests a posttranscriptional mechanism for mGluR5 up-regulation in TSC2^+/−^. We find that attenuation of Mek-Erk signaling down to WT levels recapitulates the effects of MPEP in restoring rapamycin sensitivity to mGluR-LTD, and does so in a dose-dependent fashion (Figure 4). Independently of the mTOR pathway, Erk can promote protein synthesis through phosphorylation of Mnk [45],[46] as well as through signaling to p90 ribosomal S6 protein kinase (RSK) to increase S6 phosphorylation [47]. Reduction of these pathways could explain the effect of U0126 in restoring rapamycin sensitivity.

We find that MPEP prevents DHPG from activating Erk signaling in TSC2^+/−^ slices, whereas MPEP cannot prevent DHPG-mediated Erk activation in WT slices. Thus, mGluR5 is required for Erk activation by DHPG in mutant but not WT hippocampus (Figure 3). It should be noted that in the absence of DHPG, MPEP had no effect on p-Erk or p-S6 levels (Figure 3h). MPEP is an mGluR5 inverse agonist and so is able to inhibit ligand-independent activation of the overexpressed receptor [48],[49]. Therefore, we propose that the most parsimonious explanation would be that two distinct mechanisms are at play in the TSC2^+/−^ hippocampus. One mechanism drives high basal Erk phosphorylation in mutants that is not dependent on the higher expression of mGluR5. A second mechanism allows for Erk activation in the presence of DHPG that is mGluR5 dependent in TSC2^+/−^ hippocampi but not in WT. So heightened mGluR5 levels in mutants are necessary for the LTD aberrations in adult mice because MPEP restores WT like LTD. Likewise, raised Erk activity is also necessary since UO126 restores WT-like plasticity. Finally, Erk activation by ligand requires mGluR5 in mutants but not WT, since this can be suppressed by MPEP. However, the raised basal Erk phosphorylation in mutants appears to be mGluR5 independent.

We find that slices from adult TSC2^+/−^ mice have longer epileptiform discharges induced by prolonged DHPG exposure (Figure 5b). This TSC2^+/−^ epileptiform activity is reduced by pre-incubation with MPEP, rapamycin, or U0126. Interestingly, neither rapamycin nor MPEP were able to diminish ictal duration in wild-type slices, suggesting a preferential role for mGluR5-mTOR in maintaining synchronous firing duration in TSC2 mutant slices. MPEP and UO126 were able to reduce the propensity for mutant slices to display ictal burst as measured by the number of slices that showed bursting (Figure 5d), whereas rapamycin did not. The mGluR5 positive allosteric modulator CDPPB had no effect on bursting propensity or duration in adult WT or TSC2 mutant mice. Nor did it influence burst duration or ratio of ictal to interictal events (unpublished data). These observations underscore the complex nature of synchronous firing in the seizing hippocampus: mTOR is not necessary for the increased propensity for firing in TSC whereas mGluR5 and Erk signaling are, however mTOR, mGluR5, and Erk are necessary for the increased duration of synchronous firing seen in mutants. It will be of great interest to see whether slices from juvenile mutant mice also manifest greater seizure-like activity and whether mGluR5 agonists reduce or enhance this activity given the findings of Auerbach et al. and the role of mGluR5 in younger mice [27].

Behavioral deficits and perseveration are often seen in TSC patients. In the radial arm watermaze, adult mutant mice acquired the target arm at the same rate as wild-type mouse. However TSC2^+/−^ mice showed a preference for the conditioned arm upon moving the platform. This phenotype was eliminated with administration of MPEP. This result has similarities with the work of Ehninger et al., who used the same TSC2^+/−^ mice and found they displayed extended freezing after foot shock even in a novel context. Using juvenile mice, Auerbach et al. showed that learning deficits were eliminated with mGluR5 positive allosteric modulators. These findings underscore the need to study multiple behavioral phenotypes in juvenile and adult mice with both positive and negative regulators of mGluR5.

It is interesting to note that FXS, another condition associated with overactive mGluR5 function, shares many aspects of physiology and behavior with TSC. *FMR1* knockout hippocampal slices that were disinhibited with bicuculline to induce synchronous discharges had more prolonged bursting activity and this phenotype was counteracted by MPEP [38]. Additional studies in *FMR1* knockout hippocampus demonstrate that basal levels of mGluR5 are unchanged; however, Erk1 is hyperactivated in response to DHPG [50]. The Erk hyperactivation promotes overtranslation of mRNA transcripts and can be reduced via antagonism of mGluR5 or Erk signaling, yet is immune to mTORC1 inhibition with rapamycin [50]. It has been reported that *FMR1* knockout mice exhibit rapamycin-insensitive and protein synthesis-independent mGluR-LTD [51]. We observed a similar rapamycin-insensitivity after DHPG treatment, though mGluR-LTD in TSC2^+/−^ remains dependent upon protein synthesis (Figure 1e). Though subtle differences exist, the similarities suggest that TSC and fragile X mental retardation share some common underlying mechanisms. Moreover, our results, along with the *FMR1* studies mentioned, suggest a prominent and perhaps general role for mGluR5 signaling in diseases where dysregulated postsynaptic protein translation is implicated.

The current study implicates heightened mGluR5 and Erk function in TSC pathology, thereby suggesting that available mGluR5 antagonists or Erk inhibitors may serve as therapeutic agents for treating people with TSC. The findings of Auerbach et al. suggest that mGluR5 agonists can restore appropriate LTD and behavioral phenotypes when administered to juvenile mice. In contrast we find that adult LTD, epileptiform activity, and behavioral deficits are repaired with mGluR5 antagonists. In agreement with our findings that Erk inhibition also restores appropriate LTD and suppresses epileptiform activity in TSC2 mutant slices, Chevere-Torres et al. describe normalization of LTD using U0126. In aggregate, these findings suggest that there may be an age-dependent effect of mGluR5 antagonists and agonists in the potential treatment of TSC patients. In summary we show that modulation of mGluR5 and Erk signaling can restore appropriate signaling in a disease model originating from a congenital defect, which implies that symptomatic alleviation in human TSC is possible with drugs that target these pathways.

## Methods

All animal procedures were performed with the approval of the University of Wisconsin–Madison School of Medicine and Public Health Institutional Animal Care and Use Committee and according to national guidelines and policies.

### Drugs

DHPG, MPEP, and anisomycin were purchased from Tocris Bioscience and were solublized in MilliQ water. 1,4-diamino-2,3-dicyano-1,4-bis[2-aminophenylthio]butadiene (U0126) and rapamycin were purchased from Sigma-Aldrich and dissolved in DMSO. Metformin and adenine 9-β-D-arabinofuranoside (ara-A) were purchased from Sigma-Aldrich and dissolved in MilliQ water.

### Electrophysiology

Methods were previously described in detail [30]. All electrophysiology was performed on male 21-d-old or 2-mo-old TSC2^+/−^ and wild-type littermate mice (C57BL/6 background). Individual field EPSPs were recorded with a sampling rate of 100 kHz from CA1 *stratum radiatum*, with ACSF-filled recording electrodes (1.4–2 MΩ). Baseline synaptic transmission was assessed for each individual slice by applying gradually increasing stimuli (0.5–20 V, 25 nA–2.0 µA, A-M Systems model 2200 stimulus isolator, Carlsborg, WA) to determine the input–output relationship. All subsequent experimental stimuli were set to an intensity that evoked 50% of maximum fEPSP slope.

#### LTD induction

LTD was induced with bath application of 50 µM S-DHPG for 10 min. To account for differences in recording rigs (flow rate, slice placement, etc.), the moment that slices began to demonstrate synaptic depression was set as t = 0 for all analyses. Slice recordings whose baselines deviated greater than 10% were not included. Synaptic efficacy was continually monitored (0.033 Hz).

#### Induction of epileptiform activity

Slices were prepared as described above and exposed to 50 µM S-DHPG for 30 min or DHPG with 40 µM MPEP, 20 µM U0126, or 20 nM rapamycin. All drugs were applied 10 min prior to DHPG addition, and U0126 and rapamycin were allowed to remain for an additional 10 min following removal of DHPG. Slices were then transferred to the recording chamber and allowed to recover for 1 h. Following recovery, spontaneously occurring activity was monitored in CA3 stratum pyramidale with an extracellular recording electrode. The frequency and duration of activity was characterized as interictal if less than 2 s and ictal if greater than 2 s. The proportion of slices demonstrating spontaneously occurring activity was compared between wild-type and TSC2^+/−^ mice under various drug treatments.

### Tissue Homogenization and Western Blotting

Methods were previously described [30]. Briefly, following drug application and/or stimulation, slices were flash frozen in eppendorf tubes on dry ice. Acutely harvested slices were flash frozen immediately after slicing. Following purification, protein extracts were loaded at 20–30 µg/lane in gradient (4–20%) SDS-PAGE gels (BioRad) and resolved with standard electrophoresis in tris-glycine running buffer (100 mM Tris, 1.5 M glycine, 0.1% SDS) and transferred at 4°C onto PVDF membranes and blocked in 5% milkfat TBST. All primary antibodies were applied overnight at 4°C and were obtained from Cell Signaling (Danvers, MA), except for βIII tubulin, which was obtained from Promega (Madison, WI). Membranes were the washed and incubated for 1 h in horseradish peroxidase-conjugated goat anti-rabbit IgG or goat anti-mouse IgG secondary antibodies (1∶10,000) (Santa Cruz Biotech). Protein bands were detected using SuperSignal West Femto ECL reagent (Pierce Biochem) and visualized using a UVP ChemiDoc-it Imaging System with VisionWorks software, which was also used to quantify protein bands.

### Immunoprecipitation of mTORC1 From Hippocampal Tissue

Hippocampal tissue was homogenized in ice-cold CHAPS lysis buffer (in mM) (150 NaCl, 40 HEPES, 2 EDTA, 10 pyrophosphate, 10 glycerophosphate, 4 orthovanadate, 0.3% CHAPS). Following quantification, equal amounts of cell extract were used for immunoprecipitation with 0.5 µg anti-mTOR antibody. For sham, an antibody (0.5 µg) specific to the unrelated protein LIN28b (Cell Signaling) was used. Samples were incubated on a rotator at 4°C for 2 h. We used 20 µg of whole cell extract for quantitative comparisons. We added 20 µg of washed Sepharose G beads to each sample and spun on the rotator for an additional hour at 4°C. Samples were spun down briefly, washed, and combined with SDS gel loading buffer.

### RAWM for Mice

The protocol has been described in detail [52]. Briefly, the RAWM consisted of a 2-d training protocol with 15 trials per day. Animals were injected intraperitoneally with MPEP or PBS at 30 µg/g body weight 30 min prior to training. On day 1, the animals were trained in the visible platform task first (Trials 1–9), then trained on the hidden platform version of the maze (Trials 10–15). All trials on day 2 utilized the submerged/hidden platform (Trials 16–30). The number of errors (arm entries that did not result in finding the platform) was recorded. Data were collected with VideoTrack v2.5 (ViewPoint Life Sciences Inc., Montreal, Canada).

#### Reversal training

On day 3, animals received six additional trials using a hidden platform that was moved to a different location to learn (reversal training). The new goal arm was two arms away from the location in days 1 and 2. After the hidden platform training was completed, the radial arms were removed and an open pool task with the visible platform was performed to confirm that the deficits found in the RAWM were not caused by vision or motor performance deficits in the mice. This task consisted of 5 trials, 60 s each.

#### Open field task

The open field container used measured 40 cm×40 cm×30 cm. Mice were allowed to explore the arena freely and movement was recorded in 30 s bins using VideoTrack v2.5 (ViewPoint Life Sciences Inc, Montreal, Canada). Locomotor ability was assessed by the distance (cm) ambulated during the 15-min trial. Anxiety-like behavior and exploratory drive were quantified as the percentage of distance traveled in the center of the arena versus the entire open field.

### Statistical Analyses

For electrophysiological experiments, two-way ANOVA with repeated measures (mixed model) and Bonferroni posttests were used for statistical analysis. For Western blot analysis with two sets of data, two-tailed Student *t* tests were used. Western blot analysis where multiple groups were acquired and analyzed together and one-way ANOVA with Tukey-Krameŕ posttest correction for multiple analyses were used to address significant differences between groups. Chi-square test for trend was used to analyze the contingency data obtained from the epileptiform bursting experiments. For all tests, *p*<0.05 was considered statistically significant. For behavior, two-way repeated-measures ANOVA with three-trial bins as the repeated measure was used to compare the time course for errors in RAWM. In examining individual time points, one-way ANOVA was used. Data were analyzed using Prism 5 (Graphpad Software Inc., La Jolla, CA) and all data are expressed as means ± SEM.

## Supporting Information

Figure S1 RAWM acquisition. WT and TSC2^+/−^ mice perform the task equally well during the acquisition phase. Trials 1–30: *F*_(3,27)_ = 1.44, *p* = 0.1429.
(TIF)

Figure S2 The AMPK activator 2DG (10 mM) significantly reduces mTOR-dependent mGluR-LTD in WT slices [89.6±7.1%, *n* = 7(4)] [two-way ANOVA; *F*(1, 14) = 13.84, *p* = 0.0023] and is counteracted by the AMPK inhibitor, compound C (1 µM) [58.4±4.3%, *n* = 6(3)].
(TIF)

## Abbreviations

2DG: 2-Deoxy-D-Glucose
AMPK: AMP activated protein kinase
ANOVA: analysis of variance
ara-A: adenine 9-b-D-arabinofuranoside
CA1: Cornu Ammonis 1
CA3: Cornu Ammonis 3
CHAPS: 3-[(3-cholamidopropyl)dimethylammonio]-1-propanesulfonate
DHPG: dihydroxyphenylglycine
EDTA: ethylenediaminetetraacetic acid
ERK: extracellular signal-regulated kinase
FKBP12: FK506 binding protein 12
FMR1: Fragile X mental retardation 1
FXS: Fragile X Syndrome
HEPES: 4-(2-hydroxyethyl)-1-piperazineethanesulfonic acid
LTD: long-term depression
LTP: long-term potentiation
MAPK: mitogen activated protein kinase
MEK: MAPK/Erk kinase
mGluR5: metabotropic Glutamate Receptor 5
MNK1: MAPK-interacting kinase 1
MPEP: 2-Methyl-6-(phenylethynyl)pyridine hydrochloride
mTOR: mammalian/mechanistic Target of Rapamycin
PI3K: phosphoinositide-3-kinase
RAPTOR: regulatory associated protein of mTOR
RAWM: radial arm water maze
rpS6: Ribosomal Protein S6
rpS6K: Ribosomal Protein S6 kinase
RSK1: Ribosomal S6 Kinase 1
TSC: tuberous sclerosis complex
WT: wild type
