# Supplementary figures and images for: Reduced Juvenile Long-Term Depression in Tuberous Sclerosis Complex Is Mitigated in Adults by Compensatory Recruitment of mGluR5 and Erk Signaling
Source: PLoS Biol. 2013 Aug 13;11(8):e1001627. doi: 10.1371/journal.pbio.1001627 (PMC3742461; doi:10.1371/journal.pbio.1001627)

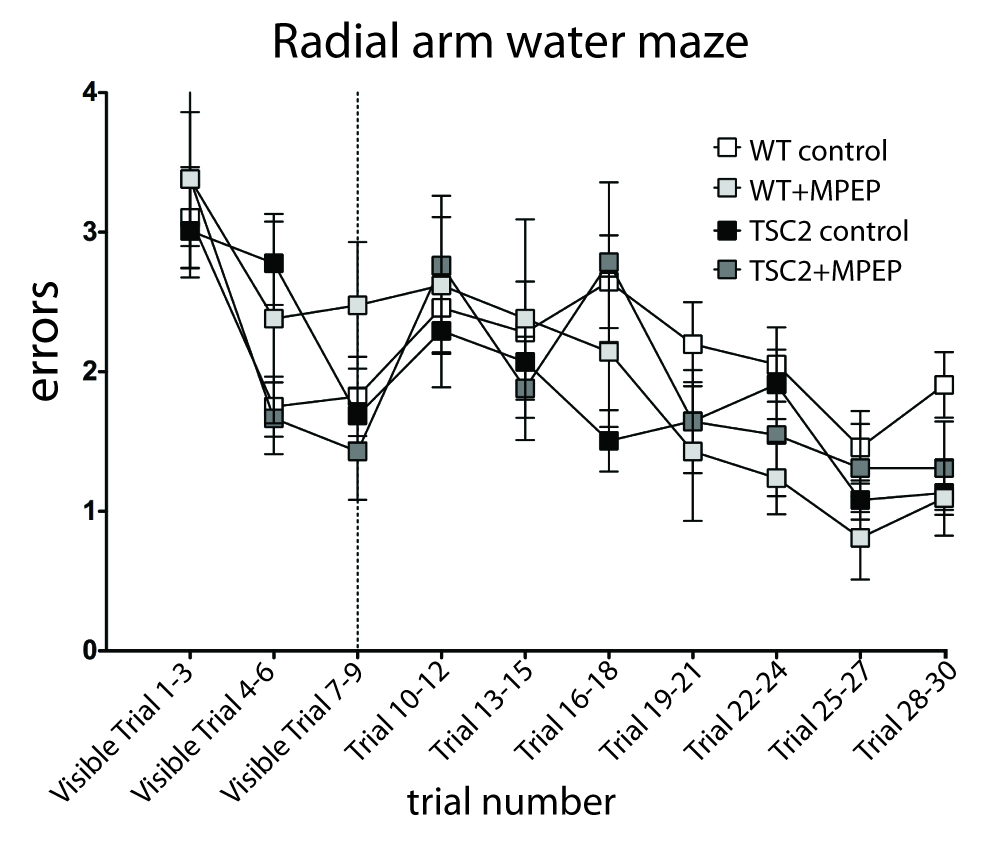

Supplement: Figure S1 — RAWM acquisition. WT and TSC2+/− mice perform the task equally well during the acquisition phase. Trials 1–30: F (3,27) = 1.44, p = 0.1429. (TIF) [file pbio.1001627.s001.tif]

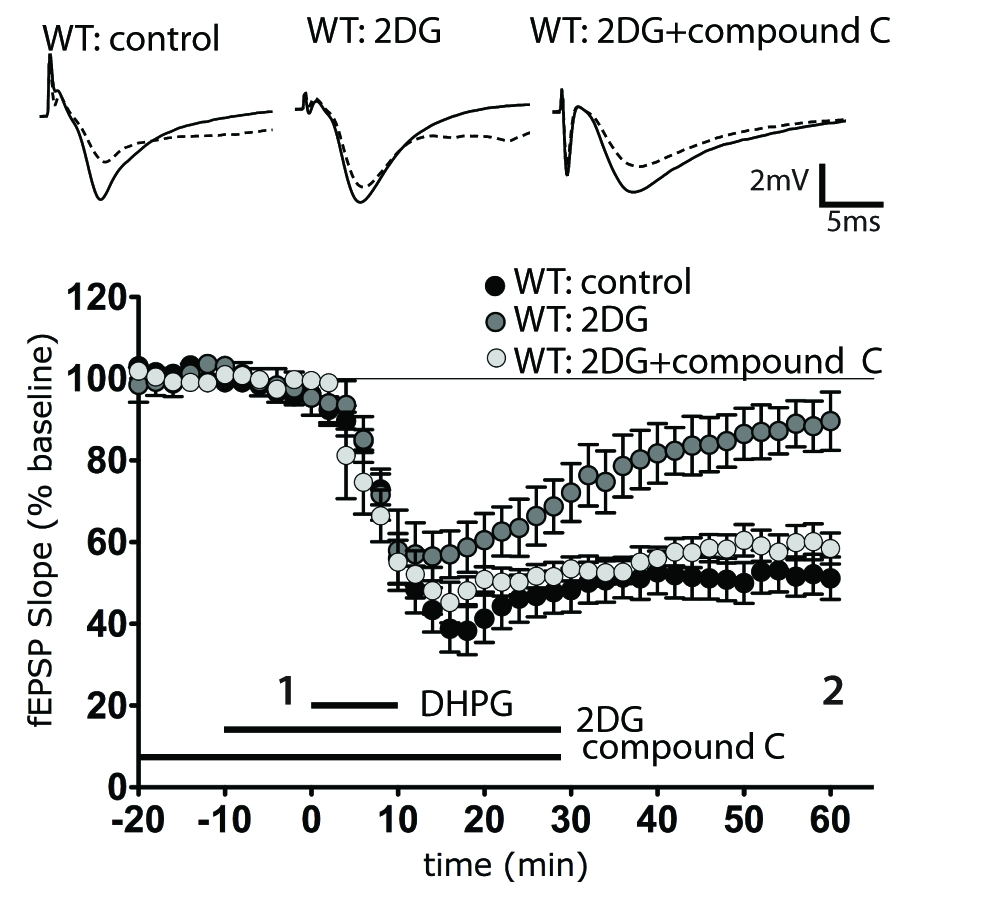

Supplement: Figure S2 — The AMPK activator 2DG (10 mM) significantly reduces mTOR-dependent mGluR-LTD in WT slices [89.6±7.1%, n = 7(4)] [two-way ANOVA; F(1, 14) = 13.84, p = 0.0023] and is counteracted by the AMPK inhibitor, compound C (1 µM) [58.4±4.3%, n = 6(3)]. (TIF) [file pbio.1001627.s002.tif]
